# Characterization of the Altered Gene Expression Profile in Early Porcine Embryos Generated from Parthenogenesis and Somatic Cell Chromatin Transfer

**DOI:** 10.1371/journal.pone.0091728

**Published:** 2014-03-14

**Authors:** Chi Zhou, John Dobrinsky, Stephen Tsoi, George R. Foxcroft, Walter T. Dixon, Paul Stothard, John Verstegen, Michael K. Dyck

**Affiliations:** 1 Department of Agricultural, Food and Nutritional Science, University of Alberta, Edmonton, Alberta, Canada; 2 International Center for Biotechnology, Minitube of America, Mount Horeb, Wisconsin, United States of America; Wellcome Trust Centre for Stem Cell Research, United Kingdom

## Abstract

The *in vitro* production of early porcine embryos is of particular scientific and economic interest. In general, embryos produced from *in vitro* Assisted Reproductive Technologies (ART) manipulations, such as somatic cell chromatin transfer (CT) and parthenogenetic activation (PA), are less developmentally competent than *in vivo*–derived embryos. The mechanisms underlying the deficiencies of embryos generated from PA and CT have not been completely understood. To characterize the altered genes and gene networks in embryos generated from CT and PA, comparative transcriptomic analyses of *in vivo* (IVV) expanded blastocysts (XB), IVV hatched blastocyst (HB), PA XB, PA HB, and CT HB were performed using a custom microarray platform enriched for genes expressed during early embryonic development. Differential expressions of 1492 and 103 genes were identified in PA and CT HB, respectively, in comparison with IVV HB. The “eIF2 signalling”, “mitochondrial dysfunction”, “regulation of eIF4 and p70S6K signalling”, “protein ubiquitination”, and “mTOR signalling” pathways were down-regulated in PA HB. Dysregulation of notch signalling–associated genes were observed in both PA and CT HB. TP53 was predicted to be activated in both PA and CT HB, as 136 and 23 regulation targets of TP53 showed significant differential expression in PA and CT HB, respectively, in comparison with IVV HB. In addition, dysregulations of several critical pluripotency, trophoblast development, and implantation-associated genes (*NANOG*, *GATA2*, *KRT8*, *LGMN*, and *DPP4*) were observed in PA HB during the blastocyst hatching process. The critical genes that were observed to be dysregulated in CT and PA embryos could be indicative of underlying developmental deficiencies of embryos produced from these technologies.

## Introduction

The domestic pig is not only an economically important livestock species, but is also an increasingly recognized biomedical animal model. Therefore, the *in vitro* production of early porcine embryos is of particular scientific and economic interest. Embryos produced from *in vitro* based systems using Assisted Reproductive Technologies (ART) are generally less developmentally competent in comparison with *in vivo* embryos. In swine, the *in vitro* production of pre-implantation embryos is much less efficient than in many other mammalian species (such as cattle) [1]. *In vitro* ART manipulations could have perturbing effects on embryonic gene expression, which potentially results in important negative long-term consequences [2], without displaying significant changes in the embryos' pre-implantation morphological characteristics [3]–[5].

Somatic cell nuclear transfer (SCNT) is a technology with great potential applications in basic and biomedical researches. However, the application of SCNT is limited by its low embryonic survival rate and the high incidence of abnormalities in individuals that develop to term, and are believed to be associated with the incorrect or incomplete nuclear reprogramming [6], [7]. Somatic cell chromatin transfer (CT) is a cloning technology that was designed to facilitate the reprogramming process [4], [8], which involves *in vitro* remodelling of the donor nuclei prior to their transfer into enucleated oocytes to remove nuclear components that may interfere with nuclear remodelling [8]. Although promising results have been reported using chromatin transfer (CT), the CT-derived embryos still exhibit abnormalities similar to those observed following conventional SCNT [7], [8]. Embryos derived from parthenogenetic activation (PA) are valuable for studies on gene imprinting [9] and are a potential alternative source of embryonic stem cells [9], [10]. However, embryos generated from PA experience severe development failure [11]. The molecular mechanisms behind the deficiencies of embryos generated from PA and CT are not completely understood.

The blastocyst is an embryonic stage that is frequently transferred into female recipients after ART manipulation [12], [13] in pig, and is, therefore, of particular scientific and economic interest. Similar to most mammalian species, the porcine blastocyst stage embryo has a distinct morphological structure that consists of the inner cell mass (ICM), internal cavity (blastocoele), and a single layer of epithelial trophectoderm (TE) with, or without (after hatching), the protective zona pellucida [14], [15]. Blastocyst stage embryos need to hatch from the zona pellucida before implantation. Following blastocyst formation, the embryo expands in size and hatches from the zona pellucida to become a “free floating” hatched blastocyst in the uterus approximately 5–6 days after fertilization [16]. This “hatching” process is a critical and tightly regulated event during early embryonic development and any dysregulation of the hatching process leads to implantation failure and results in early embryonic loss [17].

In the present study, comparative transcriptomic analyses of *in vivo* (IVV) expanded blastocysts (XB), IVV hatched blastocyst (HB), PA XB, PA HB, and somatic cell chromatin transfer (CT) HB were performed using a custom microarray platform enriched for genes expressed during early embryonic development - EmbryoGENE Porcine Array Version1 (EMPV1, NCBI Gene Expression Omnibus (GEO): GPL14925) [18].

The objectives of the present study were (1) to characterize the effect of somatic cell chromatin transfer (CT) and parthenogenetic activation (PA) on the gene expression patterns of HB stage porcine embryos; (2) to identify critical genes and gene networks that were dysregulated during blastocyst hatching in PA embryos.

## Materials and Methods

### Animal ethics statement

All animal studies were conducted in accordance with the Canadian Council on Animal Care (CCAC) Guidelines and Policies with approval from the Animal Care and Use Committee: (Livestock) for the University of Alberta (Permit Number: DYCK-2006-56).

### Recovery of in vivo embryos

*In vivo* (IVV) derived porcine Germinal vesicle (GV), MII, 2-cell (2C), 4-cell (4C), 8-cell (8C), morula(M), early blastocyst (EB), expanded blastocyst (XB), hatched blastocyst (HB) and embryonic day 11 (D11) HB (hatched blastocyst before elongation) stage embryos were collected from gilts as described previously [19] and stored individually. The day of artificially insemination is considered day 0 (D0). All embryo samples were placed on dry ice immediately after collection and stored at −80°C until RNA extraction.

### Production of in vitro-derived embryos

All of the *in vitro* (somatic cell nuclear transfer (CT) and parthenogenetic activation (PA)) embryos used in the present study were produced by the International Center of Biotechnology, Minitube of America, MT Horeb, Wisconsin, USA (http://www.minitube.com). In brief, the CT reconstructed embryos were produced by using the Chromatin Transfer technology [20], [21] under license from Hematech to Minitube (Verona, WI, USA). Oocyte collection, maturation, and micromanipulation were performed following established standard operating procedures [21], [22]. The CT reconstructed embryos (for CT embryo production) and mature oocytes (for PA embryo production) were activated with incubation in 15 μM calcium ionomycin (Calbiochem, CA, USA) supplemented mNCSU23 medium (Minitube, WI, USA) and subsequently an incubation of 1.9 mM 6-dimethylaminopurine (DMAP) supplemented mNCSU23 medium following previously established procedures [22]. The *in vitro* activated CT reconstructed embryos and the parthenogenetically activated oocytes were both cultured in the PorcPRO mNCSU-23 (Minitube, WI, USA) pig embryo culture medium system in 38.7°C, 5% CO_2_, and 95–98% humidity for up to 8 days for expanded blastocyst and hatched blastocyst development.

All of the *in vitro* (CT and PA)-derived embryo samples were placed on dry ice immediately after collection and stored at −80°C until RNA extraction.

### Total RNA isolation

Total RNA was extracted from pools of 5 embryos using Arcturus PicoPure RNA Isolation Kit (Applied Biosystems, CA, USA). The RNA quality and integrity of each total RNA sample was evaluated by Bioanalyzer RNA 6000 Pico LabChip (Agilent Technologies, ON, Canada). Except for samples from MII, 2C, 4C, and 8C stage embryos, only high quality RNA samples (RNA integrity number (RIN) ≥7.5) were used for subsequent RNA amplification. It has been demonstrated that embryos from pre-embryo genome activation (pre-EGA) stages contains very low amounts of 28S rRNA which results in lower total RNA RIN value [23]. Therefore, total RNA samples from MII, 2C, 4C, and 8C stages with lower RIN values (range from 5.8 to 6.8, clear 18S and 28S bands with no visual evidence of degradation) were utilized in this study.

### Microarray experimental design

The comparative transcriptomic analyses were performed using a custom designed porcine embryo-specific microarray platform (EMPV1: EmbryoGENE Porcine Array Version1 [GPL14925]) [18].

To characterize the effects of *in vitro* manipulations (PA and CT) on the porcine blastocyst transcriptome, comparative transcriptomic analyses among *in vivo* XB, *in vivo* HB, PA XB, PA HB, and CT HB were performed. Total RNA samples extracted from pools of 5 embryos from the same stage were amplified, labelled with Cy5 dye, and hybridized with a Cy3 dye-labelled reference amplified RNA (aRNA) pool on EMPV1 microarray following a reference design [24]–[26] using three biological replicates from each group.

Agilent two-colour RNA Spike-In (Agilent Technologies, ON, Canada) were amplified, labelled and utilized as positive controls in each hybridization reaction as previously described [18].

### Reference amplified RNA (aRNA) pool generation

A reference aRNA pool was generated from10 different embryonic stages (GV, MII, 2C, 4C, 8C, M, EB, XB, HB, and D11 HB). Total RNA samples were amplified individually using RiboAmp HS^Plus^ kit (Applied Biosystems, CA, USA); 1 ng of total RNA was used in each amplification and the quality and quantity of each aRNA sample was assessed using Bioanalyzer RNA 6000 Nano LabChip (Agilent Technologies, ON, Canada) and Nanodrop ND-1000 (NanoDrop Technologies, Wilmington, USA). A total of 360 μg of reference aRNA was generated by pooling 36 μg amplified aRNA from each of the 10 embryonic stages. The reference aRNA pool was stored in aliquots at −80°C until use.

When applied to the EMPV1 platform, the reference aRNA pool produced reference signals (signals that were higher than the average signal of negative controls) for 95% of all the genes spotted on the microarray.

### RNA amplification and labelling for microarray analysis

Due to the low quantities of each total RNA sample, all RNA samples were amplified using RiboAmp HS^Plus^ kit (Applied Biosystems, CA, USA) following the manufacturer's instructions and generated amplified RNA (aRNA) targets for microarray reactions. One ng total RNA was utilized in each amplification reaction, and the quantity and quality of the aRNA products from RNA amplification reactions were evaluated by the Nanodrop ND-1000. Two μg of aRNA were used in each labelling reaction. All labelling reactions were performed using the ULS Fluorescent Labelling Kit (Kreatech Diagnostics, Amsterdam, Netherlands) following the manufacturer's instructions. The labelling of aRNA targets was processed under an ozone-free environment. Probe concentration and labelling efficiency of each labelled sample was evaluated using Nanodrop ND-1000.

### Microarray hybridization, washing and data acquisition

The hybridization, washing and drying steps of EMPV1 microarray were conducted following the procedure described previously [18]. In short, aRNA samples were labelled with different dyes (Cy5 or Cy3) and hybridized on one microarray. Arrays were then incubated at 65°C with rotation at 10 rpm for 17 hours. After washing and drying steps that strictly followed the instructions in Agilent manual, microarrays were immediately scanned using an Axon 4200AL scanner (Molecular Device, Sunnyvale, USA). The microarray results were submitted to NCBI Gene Expression Omnibus (GEO) Database (GSE48292).

Microarray data were analysed using the FlexArray software package, which uses R and Bio-Conductor [27] and provides a user-friendly interface that facilitates data processing, visualization, and statistical analysis (Michal Blazejczyk, Mathieu Miron, Robert Nadon (2007). FlexArray: A statistical data analysis software for gene expression microarrays. Genome Quebec, Montreal, Canada, URL http://genomequebec.mcgill.ca/FlexArray). Simple background subtraction and within-array global loess normalization was performed on raw data from each array using the FlexArray software package. The threshold for positive spot selection from microarray data was determined as the mean value of all the negative control spots plus two standard deviations [18]. To identify differentially expressed genes, the normalized microarray data was analyzed using the “limma” package [28] of Bio-conductor through FlexArray under the Benjamini and Hochberg false discovery rate (BH-FDR) [29] multiple comparison correction condition through FlexArray [30]. For any particular comparison, only genes with a BH-FDR adjusted P value (B-H P-value) ≤ 0.05 and a fold change (FC) ≥ 2 (or ≤0.5) were considered to be significantly up- or down-regulated.

### Gene expression data analysis

Expression data obtained from the comparative transcriptomic analysis were analysed using the IPA (Ingenuity Pathway Analysis, Ingenuity Systems, www.ingenuity.com) Biological Functions Analysis, Canonical Pathway Analysis, and Upstream Regulator Analysis tools. The biological functions and canonical pathways analyses were performed under BH-FDR multiple testing correction conditions. Only the biological functions and canonical pathways with a BH-FDR corrected P-value (B-H P-value) <0.05 were considered significant. IPA Upstream Regulator Analysis predicts the activation status of the upstream regulator by calculating a regulation Z-score and an overlap P-value, which were based on the number of known regulation target genes from the dataset of interest, expression changes of these target genes, and their agreement with literature findings. Upstream regulators with an overlap P-value ≤ 0.05 and an IPA activation Z-score ≥ 2.0 (or ≤ −2.0) were considered significantly activated (or inhibited). Description of the calculation of the IPA regulation Z-score and overlap P-value is available in IPA white papers “A Novel Approach to Predicting Upstream Regulators”. A full description of IPA analysis is available on the IPA website (http://www.ingenuity.com) under “Upstream Regulator Analysis”, “Biological Functions Analysis”, and “Ingenuity Canonical Pathways Analysis”.

### Real-time Quantitative PCR verification of gene expression results

Fourteen genes selected from the comparative gene expression data were evaluated using SYBR Green I-based Real-time Quantitative PCR (QPCR). The primer sequences for all target genes are listed in Table 1. A total of 1 ng total RNA isolated from each pool of 5 embryos was reverse transcribed into cDNA using a high capacity reverse transcriptase (SuperScript VILO cDNA Synthesis Kit, Invitrogen) following the manufacturer's instructions with a 20 μl reverse transcription (RT) reaction volume. An equal amount (10000 copies) of a synthetic RNA transcript (Xeno RNA Control, SYBR Green Cells-to-CT Control Kit, Ambion) was added to each reverse transcription reaction to serve as an external reference for SYBR Green I-based QPCR analysis, and as a positive control for reverse transcription, in order to assess variability resulting from any RT or PCR inhibitors. The cDNA products were then diluted 5 times, and 2.5 μl of the diluted cDNA was used as the template in each of the QPCR reactions performed with StepOnePlus Real-Time PCR System (Life technologies) and Fast SYBR Green Master Mix (Applied Biosystems). The QPCR data was normalized with the external control gene (Xeno RNA Control, Ambion) using the qbase^PLUS^ software (Biogazelle) [31]. The normalized QPCR data was then further analysed using the 2^−ΔΔCT^ method [31], [32] to determine the relative differential expression (fold changes) of each target gene.

**Table 1:** Primer sequences used in Real-time PCR verification.

Gene symbol	Associated Porcine RefSeq Accession No.	Primer	Primer sequence (5'-3')	Product size (bp)
LGMN	XM_001927082	Forward	AGACGCTCCACAAACAGTAC	95
		Reverse	CAACTTCATGGCAGAGATGGA	
GATA2	NM_213879	Forward	CTCCAGCTTCACCCCTAAG	157
		Reverse	CCCGTTCATCTTGTGGTACAG	
KRT8	NM_001159615	Forward	AGATCCAAAAGCGTACCGAC	136
		Reverse	AGCTGCCTGTAGAAGTTGATC	
PSEN2	NM_001078666	Forward	CTCAACTCCGTGCTCAACA	148
		Reverse	GATGTAGGTGAAGAGGAAGAGC	
NCSTN	XM_001928786	Forward	CCCCGCAATGTCATGTTTG	92
		Reverse	AACTTGCCCTTCTCCATATCG	
HES1	NM_001195231	Forward	CTGGAGAAGGCGGACATTC	92
		Reverse	GCTCGGGTCTGTGCTTAG	
HEY2	NM_001243329	Forward	CTGCAAAGTTAGAAAAGGCCG	145
		Reverse	TCTGTTAAGCACTCTCGGAATC	
ANXA8	NM_001243599	Forward	AGACATACAAGCAGATACCAGTG	142
		Reverse	CTTCTCACCCGCTGCATAC	
SLC36A2	XM_003134141	Forward	CATCACCCAGTACATCATCCAG	127
		Reverse	CAGAACCACACCAATGCTTTC	
KCTD3	XM_003357619	Forward	AGAAGTTCCCTCTGCGAATG	149
		Reverse	CGTACCATAGGCGATCTCAATC	
NANOG	NM_001129971	Forward	GGACTTTTCCTACAATCCAGC	153
		Reverse	CCCATAAACCTCAGGCATTG	
JAG1	XM_001926559	Forward	ACATAGCCCGAAACAGTAGC	158
		Reverse	GTTGTAGCAGGGATGAGGAC	
DPP4	NM_214257	Forward	TGCGGATTCCATACCCAAAG	137
		Reverse	ATCCCCTATTAACACAGACGC	
KRT18	XM_003126180	Forward	TTGACCGTGGAGTTGGATG	149
		Reverse	ACCACTGAGGTGCTCTCC	
Xeno	Control primer Xeno from SYBR Green Cells-to-CT Control Kit (Ambion)			105

## Results

### Altered gene expression profile in PA- and CT-derived HB

The reference design, which was used in the microarray comparative transcriptomic analysis among embryos derived from the PA and CT, allows for reliable comparisons among different groups in the analysis [26], as described in the methods.

Comparative microarray analysis revealed 1492 and 103 significant differentially expressed (FC > 2 or < 0.5, B-H P-value<0.05) genes in PA- and CT-derived HB, respectively, in comparison with IVV-derived HB (Dataset S1). In comparison with IVV HB, 55 genes showed significant differential expression in both PA and CT HB, and 54 out of these 55 genes showed the same direction of expression changes (up- or down-regulation) in PA and CT HB.

**Figure 1:**
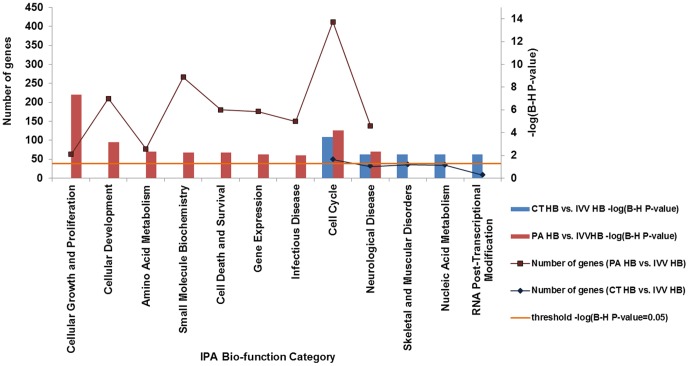
Altered biological function categories in PA and CT-derived HB. Bar chart shows the significantly altered biological function categories in IPA biological function (bio-function) analysis. Major Y axis on the left shows the number of differentially expressed genes that involved in the biological function category. Secondary Y axis on the right shows the significance (-log (B-H P-value)) of the altered biological function category. The orange line shows the significance threshold of cut off of -log (B-H P-value = 0.05).

**IPA biological function (bio-function) analysis** revealed 19 and 48 biological function categories that were significantly altered (B-H P-value<0.05, and have more than 8 molecules included in the analysis) in PA- and CT-derived HB, respectively (Dataset S2). The most significantly altered (B-H P-value<0.01, and have more than 8 molecules included in the analysis) bio-function categories in PA HB and CT HB were further identified (Figure 1). The four most significantly altered bio-functions in PA HB were associated with “cellular growth and proliferation”, “cellular development”, “cell cycle”, and “neurological disease”; and the four most significantly altered bio-functions in CT HB were associated with “cell cycle”, “neurological disease”, “skeletal and muscular disorders”, and “nucleic acid metabolism”. The “cell cycle”, and “neurological disease”-associated bio-functions were significantly altered in both PA and CT-derived HB stages embryos.

**Figure 2:**
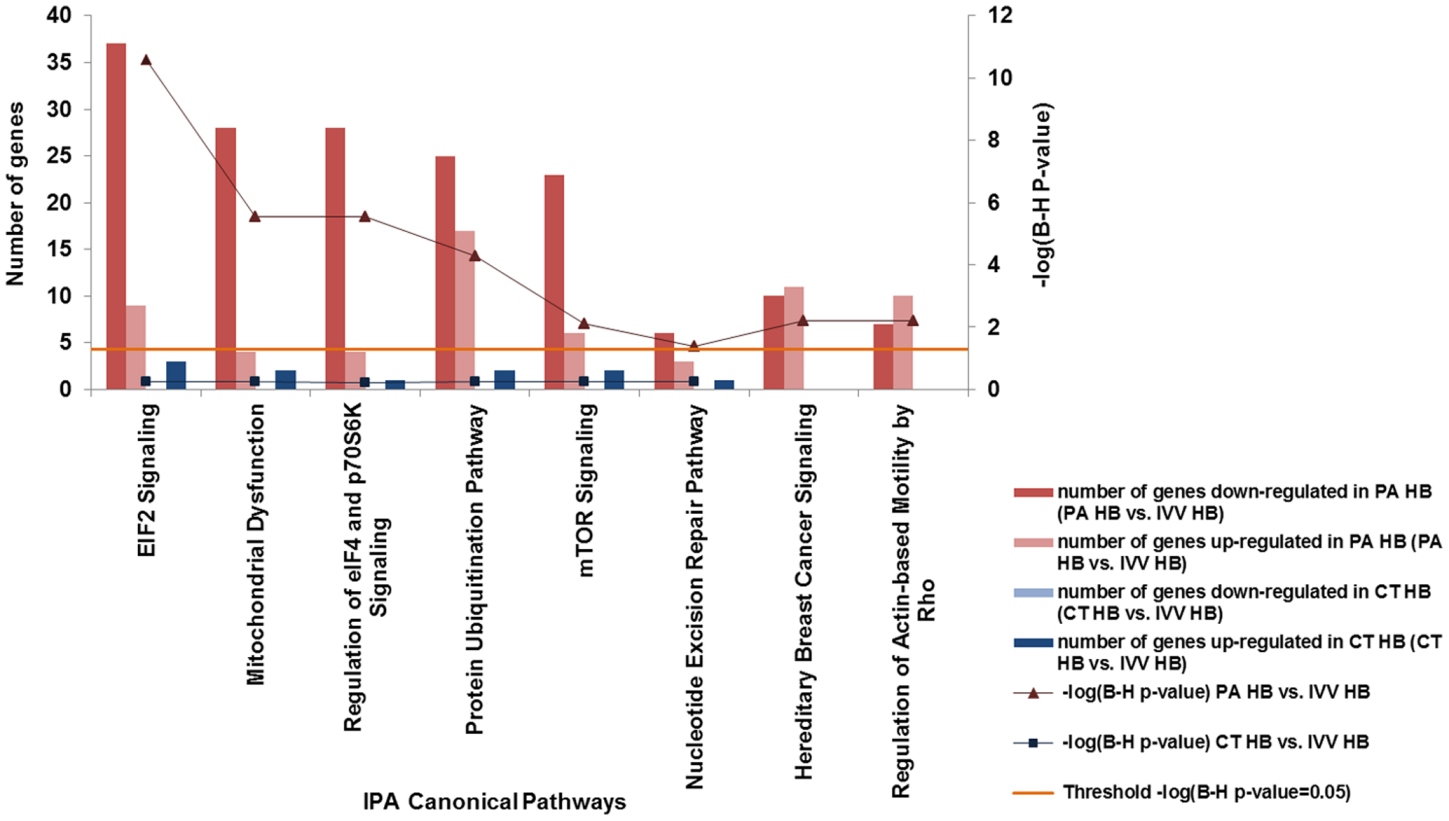
Altered canonical pathways in PA and CT-derived HB. Bar chart shows the altered canonical pathways in IPA canonical pathways analysis. Major Y axis on the left shows the number of differentially expressed genes that involved in the canonical pathway. Secondary Y axis on the right shows the significance (-log (B-H P-value)) of the canonical pathway. The orange line shows the significance threshold cut off of -log (B-H P-value = 0.05).

**IPA canonical pathway analysis** revealed eight canonical pathways that were significantly altered (B-H P-value<0.05, and have more than six molecules included in the analysis) in PA HB in comparison with IVV HB (Figure 2). The “eIF2 signalling”, “mitochondrial dysfunction”, “regulation of eIF4 and p70S6K signalling”, “protein ubiquitination”, and “mTOR signalling” pathways were the five most significantly changed canonical pathways between PA HB and IVV HB. Specifically, most of the differentially expressed genes associated with these pathways were down-regulated in PA HB (Figure 2).

**IPA upstream regulator analysis** revealed five (MYC, MYCN, NOBOX, PPARGC1A, and TP53) and one (TP53) transcription factors that predicted to be significantly activated (or inhibited) in PA and CT-derived HB, respectively, in comparison with IVV HB (Dataset S3). Transcription factors PPARGC1A, MYC, and MYCN were predicted to be inhibited in PA HB; and transcription factors NOBOX and TP53 were predicted to be activated in PA HB.

**Figure 3:**
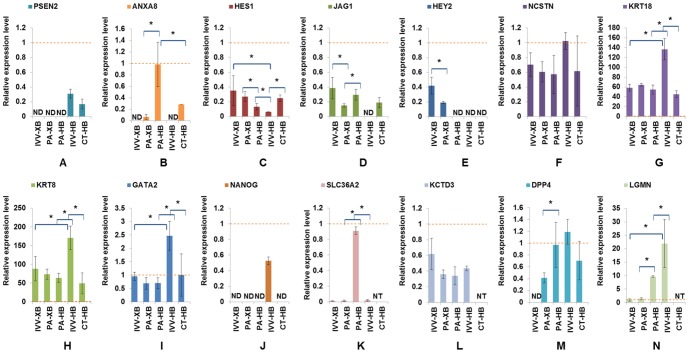
QPCR verification result. QPCR verification result of 14 selected genes. The mRNA expression levels of these genes were normalized with the external control gene (Xeno), and were calculated with 2^−ΔΔCt^ relative quantification. Bar charts showing the relative expression levels of PSEN2, ANXA8, HES1, JAG1, HEY2, NCSTN, KRT18, KRT8, GATA2, NANOG, SLC36A2, KCTD3, DPP4, and LGMN genes in IVV XB, IVV HB, PA XB, PA HB, and CT HB (KCTD3, SLC36A2, and LGMN genes were not tested in CT HB). The relative expression levels of in each sample were standardized with their expression Error bars shows the standard error (*: P < 0.05). Dashed lines indicate 1.0 expression level. ND: not detected. NT: not tested.

Transcription factor TP53 was predicted to be significantly activated in both PA and CT HB. Although no significant differential expression of the TP53 gene was observed, 136 and 23 regulation targets of TP53 showed significant differential expression in PA and CT-derived HB, respectively, in comparison with IVV embryos (Dataset S4). In addition, 11 regulation targets (*ANXA8*, *CTSH*, *CTSK*, *GSTP1*, *HSP90AA1*, *IL6*, *MYO6*, *PERP*, *PHLDA3*, *PRDX3*, and *PSEN2*) of TP53 showed differential expression in both PA and CT HB compared with IVV HB. The down-regulation of *PSEN2* (Figure 3A) and the up-regulation of *ANXA8* (Figure 3B) in PA and CT HB were confirmed by QPCR. *ANXA8* displayed detectable expression levels in both PA and CT HB, and *ANXA8* displayed significantly higher expression in PA HB than CT HB. No detectable expression of *ANXA8* was observed in IVV HB by QPCR analysis.

Significant differential expression of four “notch signalling”-associated genes (*PSEN2*, *HEY2*, *HES1*, and *J* *AG1*) were observed in PA HB in comparison with IVV-derived HB embryos. In comparison with IVV HB, the microarray analysis revealed significant down-regulation of *HEY2*, *HES1*, and *J* *AG1* genes, and significant up-regulation of *PSEN2* showed in PA HB. Significant down-regulation of the *PSEN2* genes was also observed in CT-derived HB in comparison with IVV HB. Another three (*NCSTN*, *HES1*, and *J* *AG1*) “notch signalling”-associated genes showed altered expression in CT HB in comparison with IVV HB, but with less statistical significance (FC>2 or <0.5, P-value<0.05 but B-H P-value >0.05).

Five “notch signalling”-associated genes (*PSEN2*, *HEY2*, *HES1*, *NCSTN*, and *J* *AG1*) were selected for QPCR verification. *HES1* (Figure 3C) was up-regulated in both PA and CT HB in comparison with IVV HB. *PSEN2* (Figure 3A) was down-regulated in CT HB in comparison with IVV HB, and did not display detectable expression in PA HB. *J* *AG1* (Figure 3D) did not display detectable expression in IVV HB, but was expressed in both PA HB and CT HB. *HEY2* expression (Figure 3E) was not detectable in PA, CT, and IVV HB embryos in the QPCR analysis. No significant expression change of NCSTN (Figure 3F) was observed among PA, CT and IVV-derived HB in the QPCR analysis.

In comparison with IVV HB, significant down-regulation (FC = 0.3, B-H P-value<0.05) of *KRT18* (Figure 3G) was observed in PA HB, and a less significant down-regulation (FC = 0.66, P-value<0.05 but B-H P-value>0.05) of *KRT18* was observed in CT HB. QPCR analysis of *KRT18* (Figure 3G) and *KRT8* (Figure 3H) expression showed that the *KRT18* and *KRT8* genes were down-regulated in PA HB, and the *KRT18* was down-regulated in CT HB, in comparison with IVV HB.

In addition, microarray analysis revealed significant down-regulation (FC<0.5, B-H P-value<0.05) of *GATA2* and *NANOG* in PA HB in comparison with IVV HB. QPCR analysis results confirmed this down-regulation of *GATA2* in *NANOG* in PA HB (Figure 3I and 3J). However, the down-regulation of *GATA2* in CT HB was not statistically significant. In the QPCR analysis, *NANOG* expression was only detectable in IVV HB, and no detectable expression of *NANOG* was observed in PA and CT HB.

Microarray analysis also revealed significant up-regulation (FC > 2, B-H P-value<0.05) of four precursor-microRNAs (pre-miRNA) (*MIR1343*, *MIR149*, *MIR505*, and *MIR192*) in PA HB in comparison with IVV HB. Only trends (P-value<0.05 but B-H P-value>0.05) of differential expression of the pre-miRNA of MIR505 (FC = 0.57) and MIR192 (FC = 1.57) were observed in the CT HB in comparison with IVV HB.

### Altered gene expression-regulation during blastocyst hatching of PA-derived embryos

Comparative transcriptomic analysis among IVV XB, IVV HB, PA XB, and PA HB revealed that during the transition from XB to HB, differential expression (FC > 2 or <0.5, B-H P-value<0.05) of 3 and 31 genes were observed in PA and IVV-derived embryos, respectively (Dataset S5).

The comparative microarray analysis revealed three genes (*KCTD3*, *ANXA8*, and *SLC36A2*) that showed statistically significant up-regulation from XB to HB in PA embryos. However, no significant differential expression of these three genes was observed in between IVV-derived XB and IVV HB.

QPCR analysis confirmed the up-regulation of *SLC36A2* (Figure 3K) and *ANXA8* (Figure 3B) from the XB to HB stage in PA embryos. *SLC36A2* showed no significant differential expression between IVV XB and IVV HB, and *ANXA8* expression was not detectable in IVV XB and IVV HB. No significant differential expression of *KCTD3* was observed between PA XB and PA HB in the QPCR analysis (Figure 3L).

Significant up-regulation (FC > 2, B-H P-value<0.05) of *DPP4* and *LGMN* from XB to HB in *in vivo*-derived embryos were observed in the microarray analysis. Trends toward up-regulation of the *DPP4* and *LGMN* were also observed in the PA embryos from XB to HB. In addition, a trend (P-value<0.05 but B-H P-value >0.05) of up-regulation of the trophectoderm development-associated gene *KRT8* (FC = 1.9) and *GATA2* (FC = 2.4) from XB to HB in IVV embryo was observed in the microarray analysis. However, no differential expression of *KRT8* was observed between PA XB and PA HB embryos. Results from QPCR analysis confirmed the up-regulation of *DPP4*, *LGMN*, *GATA2* and *KRT8* from XB to HB *in vivo* (Figure 3M, 3N, 3H, and 3G). In comparison with IVV embryos, *DPP4* and *LGMN* displayed a smaller up-regulation from XB to HB in PA embryos. No differential expression of *GATA2* and *KRT8* was observed between PA XB and PA HB in the QPCR analysis.

Three (*HEY2*, *HES1*, and *J* *AG1*) “Notch signalling”-associated genes showed down-regulation (FC<0.5), but with reduced statistical significance (P-value<0.05 but B-H P-value >0.05), from XB to HB in IVV embryos in the microarray analysis. *HES1* showed more than 2.5 fold down-regulation from XB to HB in both IVV and PA embryos. *HEY2* and *J* *AG1* showed more than 2.4 fold down-regulation from XB to HB in IVV embryos, but no significant differential expression of these two genes was observed in PA embryos.

Results from QPCR analysis confirmed the up-regulation of *HES1* and the down-regulation of *HEY2* and *J* *AG1* from XB to HB in IVV embryos (Figure 3C–E). Although up-regulation of *HES1* and down-regulation of *J* *AG1* from XB to HB in PA embryos were observed in the QPCR analysis, the expression changes of these two genes were less significant than IVV embryos (Figure 3C–D). HEY2 displayed a higher expression in IVV XB than PA XB, and *HEY2* expression was not detectable in both PA and IVV HB in the QPCR analysis (Figure 3E).

## Discussion

The embryos generated after *in vitro* manipulations such as parthenogenetic activation and nuclear transfer displayed slower and less effective development [33]–[36], and dysregulation of critical gene networks is probably associated with these deficiencies.

The first objective of the present study was to characterize the effects of somatic cell chromatin transfer (CT) and parthenogenetic activation (PA) on the gene expression patterns of hatched blastocyst stage porcine embryos.

Comparative microarray analysis revealed 1492 and 103 significantly differentially expressed genes in PA and CT-derived HB, respectively, in comparison with IVV-derived HB. This large gene expression profile differences between PA HB and IVV HB observed in the present study is consistent with previous studies in different species [9], [33]–[35]. The gene expression profile differences between CT and IVV-derived HB observed in the present study was less pronounced than the differences previously reported between SCNT and IVV-derive porcine blastocyst stage embryos [36].

In comparison with IVV HB, the“eIF2 signalling”, “mTOR signalling”, “regulation of eIF4 and p70S6K signalling”, “mitochondrial dysfunction”, and “protein ubiquitination pathway” pathways were the 5 most significantly altered pathways in PA HB, and most of the differentially expressed genes associated with these 5 pathways were down-regulated in PA HB.

Eukaryotic translation initiation factor 2 (eIF2) plays a key role in the recognition of the correct start codon during translation initiation process [37]. Phosphorylation of eIF2 reduces global translation and activates the transcription of “stress recovery” genes in response to environmental stresses such as amino acid deficiency, heavy metal toxicity, and bacterial infection [37], [38]. It has been reported that cells with defective eIF2 signalling were more susceptible to bacterial invasion [38]. The “mTOR signalling pathway” plays a critical role in the regulating of cell growth, proliferation, translation, protein synthesis and survival [39]–[41]. The eIF4 initiation factors are responsible for recruiting mRNA to a ribosome during translation process [42]. The translation eIF4 initiation factors and p70 S6 kinase (p70S6k) both play critical roles in the translation and protein synthesis regulation, and both eIF4 and p70S6k are regulation targets of mTOR [41], [42]. Many environmental stimuli including growth factors, hormones, and nutrient availability can regulate the eIF4 and p70S6K through “mTOR signalling pathway” [39]. The down-regulation of genes associated with the “eIF2 signalling”, “mTOR signalling”, “Regulation of eIF4 and p70S6K signalling” pathways suggest that the general translation and protein synthesis are affected in PA HB; and many “mTOR signalling”-associated critical biological processes are also significantly affected in PA HB.

Mitochondria, especially as an ATP generation source, are critical for the development of early embryos, and perturbation in their functions is associated with compromised embryonic competence [43]. Mitochondrial dysfunction in oocytes is directly responsible for the high levels of developmental retardation and early arrest of pre-implantation embryos produced *in vitro* [44]. In the present study, the down-regulation of “mitochondrial dysfunction”-associated genes in PA HB suggests compromised mitochondria function in PA HB.

The “Ubiquitin–proteasome pathway” is responsible for the selective degradation of soluble cellular proteins in most cases [45]. Ubiquitination of cellular protein is essential for the ubiquitin-proteasome pathway-dependent cellular protein degradation [46]. Degradation of maternal proteins through the “ubiquitin–proteasome pathway” is believed to be important for the oocyte-to-embryo transition [47]. In this study, significant differential expressions in genes associated with “protein ubiquitination pathway” were observed, suggesting an altered protein degradation process in PA embryos.

TP53 (tumor protein p53) encoding a well- known cell-cycle regulator and apoptosis mediator [48], and it has been previously reported that the embryos derived from parthenogenetic activation experience a higher apoptotic cell death rate [11]. Results from the present study showed that the TP53 is predicted to be activated in both PA and CT HB in comparison with the IVV HB, where the number of differentially expressed TP53 regulation targets in PA HB was more than four times higher than the number of differentially expressed TP53 regulation targets in CT HB. In addition, *ANXA8* (annexin A8) is a member of the annexins (ANXs) family, which is a group of Ca^2+^-dependent phospholipid-binding proteins. ANXs are involved in many important biological processes including vesicle trafficking, calcium signalling, cell growth, cell cycle, and apoptosis [49]. Over expression of *ANXA8* has been reported to be associated with cancer and apoptosis [50]. In the present study, *ANXA8* displayed significantly higher expression in PA HB than CT HB, and no detectable expression of *ANXA8* was observed in IVV HB. These results suggest that an activated apoptotic process might be induced in both PA and CT derived HB, and that the activation of this apoptotic process appears to be greater in PA HB than in CT HB.

NOTCH is an important regulator of development in many animals [51], which participate in many critical biological processes including cell fate specification, differentiation, proliferation, apoptosis, migration, and angiogenesis [52]. Small perturbations in Notch activity could lead to numerous developmental defects and diseases [51]. Notch signalling is initiated through ligand-receptor interactions between neighbouring cells [52]. The NOTCH-mediated HES1 expression plays an important role in the regulation of cell fate decision [52]. In mammals, the two highly homologous presenilin genes (*PSEN1* and *PSEN2*) play important roles during early embryonic development and both of the presenilin genes are positive regulators of the “notch signalling” pathway [53], [54]. Results from the present study showed that one of the mammalian Notch ligands Jagged1 (encoded by *J* *AG1* gene) [52], [55] and two other members (*HES1* [56] and *PSEN2* [54], [57]) of “Notch signalling” pathway were significantly differentially expressed between PA and IVV-derived HB. Less dramatic differential expression of these three “notch signalling”-associated genes were also observed in the CT HB. These results suggest that the “notch signalling” pathway is dysregulated in both PA and CT HB, and this dysregulation is more significant in PA HB than in CT HB. The altered regulation in Notch signalling probably contributes to the impaired development of PA and CT-derived embryos.

As one of the key regulators of pluripotency, the transcription factor *NANOG* functions as a repressor of the extra-embryonic endoderm (ExE) or primitive endoderm (PE) cell fate [58]. In comparison with IVV HB, significant down-regulation of *NANOG* in both PA and CT HB was observed in the present study, which suggests a compromised regulation of cell fate specification and TE differentiation in PA HB and CT HB.

Transcription factor GATA binding protein 2 (*GATA2*) is expressed in trophoblast giant cells and acts as important regulator for trophoblast-specific gene expression and placental function [59], [60]. Expression of *GATA2* genes is essential for normal embryonic development in rodents [59]. Keratins 8 (*KRT8*), keratin 18 (*KRT18*) and keratins 19 (*KRT19*) are predominantly expressed in epithelial components of glandular tissues in rodents and humans [61]–[63]. Expression of keratin 8 and keratin 18/19 are expressed in TE and are essential for the integrity of a specialized embryonic epithelium (trophoblast giant cells) layer and the survival of embryos [60], [62], [63]. In the present study, *GATA2*, *KRT18*, and *KRT18* showed significant down-regulation in PA HB, but only *KRT18* showed significant down-regulation in CT HB embryos in the QPCR analysis. These results suggest impaired trophoblast development in both PA HB and CT HB, and trophoblast development in CT HB is less affected than PA HB.

Although the DPP4 (dipeptidyl peptidase 4) was reported to be differentially regulated in the CT-derived bovine day 45 placenta [7], no significant differential expression of *DPP4* was observed in PA and CT HB in the present study.

MicroRNAs (miRNA) are believed to be key regulators in pre-implantation embryonic development and differentiation [64], [65]. Recent reports suggest that the microRNA reprogramming is incomplete and inconsistent in cloned embryos [35], [66]. In the present study, microarray analysis revealed significant differential expression of four pre-miRNAs in PA HB in comparison with IVV HB. Two of these 4 pre-miRNAs showed trends of differential expression, and no statistically significant differentially expressed pre-miRNA was observed between CT and IVV HB. During pre-implantation development of embryos, dynamic synthesis and degradation of miRNAs coexists [65]. Hence the differential expression of pre-miRNA does not guarantee the differential expression of mature miRNA.

In this study, the oocyte *in vitro* maturation, *in vitro* activation, as well as the embryo *in vitro* culture processes for the CT and PA embryo generation followed exactly the same procedures. The 54 common differentially expressed genes that were observed in CT and PA embryos in comparison with IVV embryos could be associated with any of these common *in vitro* manipulation processes. Further studies are necessary before the differential expression of these “common differentially expressed genes” could be connected with any specific *in vitro* process.

The second objective of the present study was to identify dysregulated genes and gene networks in PA embryos during blastocyst hatching. Hatching is a critical and necessary process during the early development of mammalian embryos. Blastocyst hatching is a well programmed and tightly regulated event, and dysregulation of this critical process leads to implantation failure and results in early embryonic loss [17]. Dysregulation of critical genes and gene networks during blastocyst hatching process are probably contributed to the deficiencies in embryos generated from PA.

In the present study, significant differential expression of 31 genes were observed during the blastocyst hatching process (from XB to HB) in IVV embryos, but these 31 genes were not properly regulated in PA embryos during blastocyst hatching. On the other hand, *SLC36A2* and *ANXA8* showed significant up-regulation during the blastocyst hatching process in PA embryos, but no up-regulation of these two genes were observed in IVV embryos. SLC36A2 (Solute carrier family 36 (proton/amino acid symporter), member 2) mediates the transport of amino and fatty acids, which are critical to early embryonic development [67], [68]. Further work is necessary to determine if this up-regulation of *SLC36A2* is compensating for the function of other dysregulated genes in PA embryos and reflecting the increased need for nutrients in the rapidly developing embryos.

LGMN (legumain), also known as cysteine protease 1, is involved in protein processing and is highly expressed in the placenta of pig [69]. Legumain has been reported to be expressed in bovine trophoblast and associated with the regulation of trophoblast invasiveness and endometrial remodelling during implantation [70]. DPP4 (dipeptidyl peptidase 4) is a membrane-bound aminopeptidase, which is associated with placental development and the establishment of proper fetal-maternal interactions in cattle and human [7], [71]. In the present study, dramatic up-regulation of *LGMN* and *DPP4* were observed during hatching process in IVV embryos, but the expression changes of *LGMN* and *DPP4* observed during hatching process in PA embryos are much less dramatic. Results from the present study showed that the expression of several critical pluripotency, trophoblast development, and implantation-associated genes (*NANOG*, *GATA2*, *KRT8*, *LGMN*, and *DPP4*) were not properly regulated during the blastocyst hatching process in PA embryos. In addition, altered regulation of “notch signalling”-associated genes was also observed during the blastocyst hatching process in PA embryos. Failing to regulate the expression of these critical genes during the hatching process is probably contributed to the delayed and less efficient development of PA embryos.

Further protein expression level data for these dysregulated genes that were identified in the present study would be necessary before a definite link could be drawn between dysregulations of these genes and deficiencies that observed in PA and CT embryos.

## Conclusion

In the present study, we have successfully characterized the altered gene expression profiles in porcine HB embryos derived from parthenogenetic activation and somatic cell chromatin transfer, in comparison with *in vivo*-derived HB. Specifically, we have identified several signalling pathways, critical genes, and critical gene networks that were significantly altered in the PA- and CT-derived HB stage embryos. In addition, we have also identified several critical genes that were not properly regulated during the blastocyst hatching process in embryos derived from PA.

To date, morphological characteristics and blastocyst formation rate are still two of the major parameters commonly used in embryonic developmental competence assessment [2]. Results from the present study showed that embryos produced from PA and CT could develop into expanded blastocyst and hatched blastocyst stage, even with dysregulations of critical pathways and gene networks. Hence, the morphological criteria and blastocyst development ratio are insufficient to determine the ultimate competence of embryos generated after *in vitro* ART manipulations (such as PA and CT). The critical genes that exhibited altered expression in CT and PA embryos could be indicative of underlying developmental deficiencies of embryos produced from these technologies.

## Supporting Information

Dataset S1 **Expression data of significant differentially expressed (B-H P-value<0.05, FC>2 or <0.5) genes in PA HB vs. IVV HB and CT HB vs. IVV HB analyses.**
(XLSX)

Dataset S2 **IPA bio-function analysis result of significantly altered (B-H P-value<0.05, molecules involved in the analysis ≥ 8) biological function categories in PA HB vs. IVV HB and CT HB vs. IVV HB analyses.**
(XLSX)

Dataset S3 **IPA upstream regulator analysis result of transcription factors that predicted to be significantly activated or inhibited (overlap P-value<0.01, IPA Activation z-score >2 or <−2) in PA HB vs. IVV HB and CT HB vs. IVV HB analyses.**
(XLSX)

Dataset S4 **Expression data and IPA upstream regulator analysis prediction of differentially expressed (B-H P-value<0.05, FC>2 or <0.5) regulation targets of transcription factor TP53 in PA HB vs. IVV HB and CT HB vs. IVV HB analyses.**
(XLSX)

Dataset S5 **Expression data of genes that differentially expressed (FC > 2 or <0.5, B-H P-value<0.05) during the blastocyst hatching process in PA and IVV-derived embryos.**
(XLSX)
